# Supplementary material for: Medication Adherence in Hypertensive Individuals in Panama 2019: A National Cross-Sectional Study
Source: Healthcare (Basel). 2022 Nov 9;10(11):2244. doi: 10.3390/healthcare10112244 (PMC9690778; doi:10.3390/healthcare10112244)
Supplement: Supplementary file 1 [file healthcare-10-02244-s001.zip › S1 Table.pdf]

Table S1
Baseline characteristics among treated hypertensive participants according to whether they responded to the 4-items MMAS. Prevalence (%) and 95% CI (confidence intervals).

	MMAS answered N=395,086	MMAS not answered N=221,298
**Demographic Characteristics, % (95% CI)**		
Sex		
Men	44.9 (40.9–48.9)	48.6 (43.4–53.9)
Women	55.1 (51.1–59.1)	51.4 (46.1–56.6)
Age, years		
Median (IQR)	63 (53–73)	56 (43–71)
Ethnicity		
Caucasian	22.0 (18.6–25.8)	26.6 (21.9–31.8)
Afro-Panamenian	18.6 (15.7–21.9)	18.4 (14.5–23.0)
Mestizo	52.6 (48.7–56.4)	45.0 (40.0–50.0)
Indigenous	3.3 (2.4–4.5)	6.1 (4.1–9.1)
Others (including Asians)	3.5 (2.4–5.3)	4.0 (2.5–6.3)
Region		
Urban	73.0 (70.2–75.7)	74.3 (69.9–78.2)
Rural	26.0 (23.4–28.8)	24.7 (20.8–29.0)
Indigenous	1.0 (0.6–1.5)	1.1 (0.7–1.8)
**Socioeconomic Characteristics, % (95% CI)**		
Education		
Higher education	19.5 (16.0–23.4)	19.1 (15.2–23.8)
Secundary education	41.5 (37.8–45.2)	47.0 (41.8–52.3)
Primary education	34.9 (31.5–38.4)	29.3 (25.2–33.8)
No education	4.2 (3.2–5.5)	4.6 (3.1–6.7)
Individual Monthly Income (Tercile)		
≥ 500 USD	34.9 (30.7–39.2)	40.4 (34.6–46.5)
145 – 499 USD	38.2 (34.3–42.2)	40.8 (35.3–46.6)
0 – 144 USD	27.0 (23.7–30.5)	18.8 (15.5–22.6)
**Lifestyle behaviour, % (95% CI)**		
Current smoker		
Yes	3.2 (2.2–4.5)	5.8 (3.3–10.2)
BMI cathegories		
Underweight	1.4 (0.7–2.6)	1.5 (0.7–3.3)
Normal	18.2 (15.3–21.6)	23.1 (18.6–28.2)
Overweight	36.6 (32.8–40.5)	32.4 (27.5–37.8)
Obesity	43.9 (39.8–48.0)	43.0 (37.9–48.3)
High sodium food consumption		
Yes	42.4 (38.5–46.4)	43.3 (38.3–48.5)
Physical Inactivity		
Yes	52.6 (48.1–57.1)	69.1 (63.4–74.2)
Alcohol consumption		
Non-drinker	86.5 (83.4–89.1)	90.4 (86.6–93.2)
Moderate drinker	5.2 (3.7–7.4)	4.3 (2.5–7.4)
Excessive drinker	8.3 (6.2–10.9)	5.3 (3.3–8.3)
**Comorbidities, % (95% CI)**		
Self-reported medical history of CKD		
Yes	20.1 (15.4–25.9)	15.9 (13.6–18.5)
Self-reported medical history of CVD		
Yes	3.0 (1.7–5.3)	0.7 (0.2–2.6)
Self-reported medical history of AMI		
Yes	1.0 (0.6–1.8)	0.1 (0.0–0.9)
Comorbid anxiety and/or depression		
Yes	2.7 (1.8–3.9)	3.7 (1.9–7.2)
**Time from hypertension diagnosis, % (95% CI)**		
Reported taken medication in the previous two weeks of study enrollment	23.2 (19.9–26.9)	55.9 (50.8–60.9)
Previous year	10.0 (8.0–12.5)	7.8 (5.7–10.7)
1–5 years	21.1 (18.3–24.2)	16.6 (13.1–21.0)
≥6 years	45.7 (41.9–49.5)	19.6 (16.4–23.4)
**Health check-ups, % (95% CI)**		
None	11.5 (9.2–14.4)	20.5 (16.4–25.3)
1–2 yearly	55.9 (52.1–59.6)	60.4 (55.1–65.4)
≥3 yearly	32.5 (29.2–36.1)	19.1 (15.3–23.6)

MMAS: Morisky Medication Adherence Scale; N=weighted study population; CI=confidence intervals; IQR=interquartile range: USD=United States dollar. BMI=body mass index. CKD=chronic kidney disease. CVD=cerebrovascular disease. AMI=acute myocardial infarction. Missing value on MMAS (1%), education (1.7%), individual monthly income (16.6%), BMI categories (12.4%), high sodium food consumption (1.2%), and physical inactivity (25.2%).
