# Supplementary material for: Medication Adherence in Hypertensive Individuals in Panama 2019: A National Cross-Sectional Study
Source: Healthcare (Basel). 2022 Nov 9;10(11):2244. doi: 10.3390/healthcare10112244 (PMC9690778; doi:10.3390/healthcare10112244)
Supplement: Supplementary file 1 [file healthcare-10-02244-s001.zip › S2 Table.pdf]

Table S2. Checklist of items that should be included in reports of ***cross-sectional studies***

	Item No	Recommendation	Line number (using Windows)
**Title and abstract**	1	(*a*) Indicate the study's design with a commonly used term in the title or the abstract	2-3
		(*b*) Provide in the abstract an informative and balanced summary of what was done and what was found	9-32
**Introduction**			
Background/rationale	2	Explain the scientific background and rationale for the investigation being reported	35-79
Objectives	3	State specific objectives, including any prespecified hypotheses	80-83
**Methods**			
Study design	4	Present key elements of study design early in the paper	85-110
Setting	5	Describe the setting, locations, and relevant dates, including periods of recruitment, exposure, follow-up, and data collection	86-105
Participants	6	(*a*) Give the eligibility criteria, and the sources and methods of selection of participants	86-92
Variables	7	Clearly define all outcomes, exposures, predictors, potential confounders, and effect modifiers. Give diagnostic criteria, if applicable	123-157
Data sources/ measurement	8*	For each variable of interest, give sources of data and details of methods of assessment (measurement). Describe comparability of assessment methods if there is more than one group	*111-122*
Bias	9	Describe any efforts to address potential sources of bias	165-172
Study size	10	Explain how the study size was arrived at	102-105
Quantitative variables	11	Explain how quantitative variables were handled in the analyses. If applicable, describe which groupings were chosen and why	161-163
Statistical methods	12	(*a*) Describe all statistical methods, including those used to control for confounding	167-172
		(*b*) Describe any methods used to examine subgroups and interactions	172
		(*c*) Explain how missing data were addressed	163-164
		(*d*) If applicable, describe analytical methods taking account of sampling strategy	162-163
		(*e*) Describe any sensitivity analyses	NA
**Results**			
Participants	13*	(a) Report numbers of individuals at each stage of study—eg numbers potentially eligible, examined for eligibility, confirmed eligible, included in the study, completing follow-up, and analysed	172-181
		(b) Give reasons for non-participation at each stage	NA
		(c) Consider use of a flow diagram	181
Descriptive data	14*	(a) Give characteristics of study participants (eg demographic, clinical, social) and information on exposures and potential confounders	186-205
		(b) Indicate number of participants with missing data for each variable of interest	
Outcome data	15*	Report numbers of outcome events or summary measures	206-233

Main results	16	(*a*) Give unadjusted estimates and, if applicable, confounder-adjusted estimates and their precision (eg, 95% confidence interval). Make clear which confounders were adjusted for and why they were included	234-252
		(*b*) Report category boundaries when continuous variables were categorized	234-252
		(*c*) If relevant, consider translating estimates of relative risk into absolute risk for a meaningful time period	NA
Other analyses	17	Report other analyses done—eg analyses of subgroups and interactions, and sensitivity analyses	Gender-specific models
**Discussion**			
Key results	18	Summarise key results with reference to study objectives	253-256
Limitations	19	Discuss limitations of the study, taking into account sources of potential bias or imprecision. Discuss both direction and magnitude of any potential bias	291-303
Interpretation	20	Give a cautious overall interpretation of results considering objectives, limitations, multiplicity of analyses, results from similar studies, and other relevant evidence	257-290
Generalisability	21	Discuss the generalisability (external validity) of the study results	304-306
**Other information**			
Funding	22	Give the source of funding and the role of the funders for the present study and, if applicable, for the original study on which the present article is based	319-324

*Give information separately for exposed and unexposed groups.

Note: An Explanation and Elaboration article discusses each checklist item and gives methodological background and published examples of transparent reporting. The STROBE checklist is best used in conjunction with this article (freely available on the Web sites of PLoS Medicine at http://www.plosmedicine.org/, Annals of Internal Medicine at http://www.annals.org/, and Epidemiology at http://www.epidem.com/). Information on the STROBE Initiative is available at www.strobe-statement.org.
